# Practical Observations on the Uterine Hœmorrhage, Connected with Abortion and Parturition

**Published:** 1828-08

**Authors:** 


### Art. II. *Practical observations on the Uterine Hœmorrhage, connected with Abortion and Parturition.* By the Editor.

Few subjects have engaged the attention of Obstetricians, more than Uterine hœmorrhage. Without claiming to have had any uncommon share of obstetrical practice, I may say that, in attendance on many hundred patients, belonging to that department of the profession, I have been presented with almost every kind and degree of uterine hœmorrhage, and have not, in the course of more than 20 years practice, lost a single patient with that formidable malady. Was this result ascribable to new views of the pathology of that affection and a new mode of practice, it would certainly be proper to report them. I have relied, however, on remedies generally known to the profession. Ought I then to consume the time of the readers of this journal, by setting forth the plan which I have followed? I see no objection to such a publication; inasmuch as it can scarcely fail to be useful, for practitioners, occasionally, to draw monographs of their practice, even in maladies the most curable. I shall state what I have to say under three distinct heads—Prevention, Suppression and Restoration.

### I. Of Prevention.

1. When uterine hœmorrhage is expected, either, at the proper time of parturition, or before, much may be done to avert it. If the patient be of a sanguine temperament and full habit, blood-letting is indispensable; and in proportion to the extent to which it is carried, will the danger of hœmorrhage be diminished. There are, however, feeble and dyspectic constitutions, in which a resort to this preventive remedy, would be unavailing, and, indeed, prejudicial. In such constitutions, an indiscriminate use of the lancet, in the period of utero gestation, is altogether improper, and has often produced abortion.

2. Another prophylactic is purging, which, by weakening the action of the heart, diminishes the impulse into the uterine arteries; and by promoting secretion and peristaltic action, in the bowels, seems to lessen the determination to the uterus.

3. A third and most powerful means of prevention, is the liberal administration of opium or laudanum. This should follow the blood-letting and purging, when those remedies are employed. The quantity given, should be great enough to exert a crippling influence on the circulatory system, which, in vigorous constitutions, cannot be easily affected, if venesection be not premised. The power of the narcotic over a hœmorrhagic tendency is so great, that when there is no particular degree of plethora, I feel little apprehension for any patient, after being brought under their influence.

4. A recumbent posture, a hard bed and warm feet are all requisite; but can, by no means, be relied on to prevent hœmorrhage, where the tendency to it is very great. I cannot, however, too strongly insist on the necessity of perfect rest and physical and moral quiet, immediately after delivery. Many a patient has been brought into a most reduced state, simply by a neglect of these precautions.

II. Of Suppression.

1. When uterine hœmorrhage begins, whatever may have been the vigour of the circulation, it is speedily brought below the standard of health. Venesection, therefore, however valuable, as a means of prevention, is wholly inadmissible as a means of cure, except in the earliest stages.

2. But the same remark does not apply to opium, which I have found adapted to every stage of this *profluvium.* The quantity required is sometimes very great; and if it be not thus administered, but little benefit will result from its use. Two or three grains of solid opium, or a large tea-spoon full of strong laudanum, may be given at a single portion, and repeated once or twice, according to circumstances. Twenty or thirty years ago, the union of acetate of lead, and ipecacuanha, with opium, was strongly recommended, by several practitioners, especially the late professor Barton. I have often resorted to the combination; but am by no means satisfied of its superiority over uncombined opium. When employed at all, it should be in the early stages, when the sedative power of the two adjuvants may exert a favorable influence over the action of the heart and arteries. In exhausted states of the system, this influence is not beneficial, nor, even, safe.

3. The abdominal bandage, immediately after delivery, should never be neglected, when hœmorrhage is threatened. It may be promptly applied, in a temporary way, over the clothes of the patient, and readjusted after some hours. By compressing the uterus between the *parietes abdominis*, and the contained viscera, it contributes powerfully to promote the tonic contractions of that organ, so essential to the suppression of hœmorrhage.

I have heard fears expressed, that a tight bandage, immediately after parturition, might do harm; but such apprehensions are groundless. It is only the unskilful application of it that can do injury. As too often applied, it is a mere cord drawn around the patient, over the hypochondriac and epigastric regions. Such a ligature interferes with the action of the diaphragm, and by its pressure on the scrobiculus cordis, inevitably distresses the patient, while it is far from fulfilling the intention with which it is employed. The bandage should be broad and firm, and passed over the iliac, hypogastric and umbilical regions; and ought to be drawn so tight, especially below, as to compress the viscera firmly against each other, for on this all its efficacy depends. Now this compression is attended with three excellent effects: First, it maintains the excitement of the heart and brain; and, in bad cases, prevents syncope:—Secondly, it prevents or restrains hœmorrhage:—And, thirdly, it facilitates the separation and discharge of the placenta. To promote the last object, it is common to direct the patient to make long inspirations and expirations, and to employ frictions over the region of the uterus; but the former do far more good with, than without the bandage; and the latter are, in almost every respect, inferior to it. To its influence, chiefly, I ascribe the fact, that I have never yet, in my own practice, been obliged to introduce my hand into the uterus for the purpose of separating the placenta from its attachment.

In speaking of the safety of the tight bandage, it will be understood, that I refer to the period immediately after delivery; which is, in reality, the only one that imperiously requires it. At a subsequent time, when the circumstances that favor hœmorrhage no longer exist, and the sensibility of the abdominal parieties and the contained viscera, has become exalted; the tight bandage would be injurious, and is, in reference to the objects for which it was applied, quite unnecessary.

4. Cool air and cold topical applications, are of great moment; but too much reliance should not be placed on them. as long as the heart and arteries retain a considerable degree of vigour, they are highly beneficial; but in states of the system greatly reduced, if too liberally applied, they may do harm; and in every stage, it is important to preserve the temperature of the lower extremities. The injection of cold water into the cavity of the uterus, might, undoubtedly, be resorted to, in desperate cases, with success, though subsequent hysteritis might sometimes, perhaps, ensue.

5. The Tampon is a remedy, which may often produce the most salutary effects. Of the propriety of a resort to this instrument, in cases of early abortion, there can be no difference of opinion; as the uterine cavity is then, in general, so small, that it may be filled with coagula, before the patient would suffer any alarming reduction of strength. There are many practitioners, however, who condemn a resort to it, after ordinary parturition. But even then, it does good, though it should not be relied upon to the neglect of other means. That it may prevent the external discharge, while the large uterine cavity is filling with blood, no one can deny; but this need not operate to the disadvantage of the patient, unless the practitioner takes it for granted, that there is no discharge *into* the uterus, because he has prevented a discharge *from* it. He should look well to the state of the circulation, and, also, to the animal functions generally; and if he finds them failing, he will stand in little need of a vaginal discharge, to apprize him of the continued existence of hœmorrhage. Now, in such a case, will it be diminished, by withdrawing the Tampon, and suffering the blood to flow out uncoagulated? I answer that it will not; but the reverse must happen. For if a coagulum, produced by the restraining influence of the Tampon, in the early periods of gestation, can moderate the hœmorrhage, it is equally true, that it can do the same thing, after delivery, at the full period. I would, therefore, promote the formation and retention of coagula, within the uterine cavity; and rely upon their contact with the bleeding surface, pressed against them by the action of the external bandage, to moderate the discharge. At the same time, I would resort to every other remedy, as if the Tampon had not been employed. It is only objectionable, when it is made our exclusive reliance.

6. Few cases, in the profession, offer such alarming indications of exhaustion and debility, as the hœmorrhages which we are considering. And, what is more appalling, the discharge may continue, notwithstanding the powers of the heart are extremely reduced. Under such circumstances, internal and external stimulation, is of the utmost importance. To effect the former, brandy, whiskey, ammoniated alcohol, tincture of camphor, and other excitants, united with laudanum, may be administered freely; but the stomach is sometimes irritable, and oftener torpid, so that we shall, frequently, be disappointed in our attempts to sustain the sinking energies of the patient, by internal excitants. In these cases, the pulse becomes exceedingly feeble and empty, the respiration embarrassed, the surface pale, cool and sweaty, and the feet cold; but above all, there occurs an epigastric anguish, which suggests to the patient that death is approaching. Now, in this extremity, as well as in the approach to it, I am convinced that nothing is equal to external stimulation, with mustard. It not only arrests the declining powers of the system, but restrains the hœmorrhage, which is exhausting the patient. I feel quite certain, that I have saved the lives of several patients, by a resort to sinapisms. As no time is to be lost in these cases, the physician should, in general, take upon himself their preparation; and in the first place, the mustard which he uses, ought to be good. He should prefer the unpulverized seed, which may be crushed, for the occasion, and will be free from the ordinary adulterations.

Secondly, it should be mixed up with boiling water or vinegar, and used as hot as the patient can bear it. Thirdly, the napisms, thus prepared, should be applied, of a large size, to the epigastrium, and to the lower and inner sides of the legs. In extreme cases, when the feet are cold and insensible, we can only rely upon epigastric irritation; and its animating influence on the various functions, is, in all respects, admirable. The pulse rises in its volume, power and regularity; or, if entirely suspended, which occasionally happens, it is rendered perceptible; the death-like sensation of sinking and of syncope, is removed; and the hœmorrhage moderated. In such cases, it will not answer to rely upon applications to the lower extremities; for in their state of coldness and insensibility, the mustard will not act upon them, in due time. It is proper, however, to apply them, that when the epigastric sinapism may have revived the susceptibility of the surface generally, the returning excitement may be augmented and sustained by their action. When they excite pungent pain, and the patient becomes restive, under their impress, they may be removed; but in phlegmatic temperaments, their re-application will often be necessary.

By these means, varied and combined, according to circumstances, I have had more success in the treatment of uterine hœmorrhages, than I have been so fortunate as to experience, from other agents in the treatment of any other dangerous disease; and am disposed, therefore, to think highly of them. In their application, I have laid the greatest stress on opium, the tight bandage, and sinapisms; and if, in my relative estimate of the value of our different resources, I have differed, in any degree from others, whose practice I have known, it has been in pressing *these* measures with greater energy; and placing less reliance upon cold vinegar, cool air, sugar of lead, and warm irons to the feet.

After every copious uterine hœmorrhage, there succeeds a period of feverish excitement, with head-ache, and morbid vigilance. The proper remedy for this condition, is alvine evacuation; which may be effected either by enemata or a cathartic. The young practitioner is often apprehensive, however, that purging may reproduce the hœmorrhage; but of this there is no danger. He need not, therefore, suffer his patient to linger under these symptoms; but resort to the means, of which I have spoken, at an early period. They will not only afford great relief from those symptoms; but contribute equally to an abatement of the after pains, which, frequently, prove so obstinate, under the narcotic treatment. This is particularly true of emollient enemata, which will, in general, give more relief from that painful affection, than any other remedy, at present known to the profession.

### III. Of Restoration.

The recovery from copious uterine hœmorrhage, especially that connected with abortion, is generally tedious. The digestive, indeed, the nutritive organs generally, fall into torpor and depraved action. In fact, the great functions of circulation and innervation, are left more or less reduced and perverted; and their restoration is not easily effected. Stimulants, tonics, and nutrients are the means; but under an undiscerning and indiscriminate administration, it is far from being certain, that they will succeed to the satisfaction either of the patient or physician. They must be so administered, that the former do not excite headache or epigastric heat; nor the latter, acidity of the stomach. It is necessary, in short, to look diligently to the state of the great secretions, especially those of the liver, intestines, kidneys and skin. Some one or more of these will generally be found in a state of bad health, and must be treated with appropriate stimuli. Hence, it is necessary, while administering general stimulants, to employ those, also, which act upon particular organs. Thus, the secretion of bile must be maintained by the pil. hydrargyri and aloes, formed into pills with extract of gentian. Intestinal secretion and excretion, must be promoted by the same composition; by small doses of cinchona bark and jalap; or an infusion of senna, with bitters and aromatics, to which the carbonated alkalies or magnesia, may be advantageously added. The stomach must be regulated in its susceptibilities by an occasional emetic of ipecacuanha, from which the best effects will often result. The urinary secretion, when deficient, must be promoted, by stimulating diuretics. Lastly, the functions of the skin should be maintained by frictions, flannel, exercise, and the cold or warm bath. If we thus attend to the state of the secretions and excretions, our patients, debilitated by copious hœmorrhage, will seldom experience inconvenience from the most active stimulation, while its restorative influence will be decided.

There are too morbid states connected with, or rather making part of, that now under consideration, which frequently cause great suffering to the patient, and trouble to the physician;—I allude to hysteria, and prolapsus uteri with leucorrhœa. They are very commonly united. In patients of a sanguine and vigorous temperament, they, in general, yield promptly, to the treatment of which I have spoken; but such are not their usual subjects; and, attacking, chiefly, those of a phlegmatic or nervous constitution, they are apt to prove obstinate. It is not my intention to dwell upon the treatment of these symptoms; for, in fact, I know of little that can be done, save that which has been recommended. I may refer, however, to the speedy exhaustion, which the susceptibility of the system suffers, under the diffusible stimulants, which are usually employed in these cases. Thus the antispasmodic, which, to-day, afforded great tranquility to the nervous system, may entirely fail to-morrow; when another prescription will be necessary. It is not indispensable, however, to resort to *different* medicines, every time we prescribe anew, for when we employ composite recipies (which, in general, are most successful in these cases) a mere change in the proportion of their ingredients, will frequently answer the end in view.

As to the local treatment of the uterine system, I am by no means convinced, that it affords much relief. Astringent injections sometimes do good, but their effects are far too limited. From pessaries, I have seen still less benefit, and they give the patient more trouble, than injections. A pelvic cold bath is serviceable, if persevered in. Frictions and pharmaceutic stimulants over the sacral portion of the spiral column, are useful. A recumbent position on a mattrass or straw bed, is, in all bad cases, quite indispensable; when the patient is not engaged in taking exercise. Walking is prejudicial, but our chief reliance should be on carriage and horse riding. The patient will often resist the proposal to go abroad, on the ground of being too weak. But the physician must decide; and he should not be backward in urging that, which, in the majority of cases, will contribute more to allay nervous irritation and restore tone, both general and local, than the whole materia medica.

I shall conclude this series of practical remarks, (extended much beyond the limits which I expected them to reach) by a reference to the injury, which, occasionally, happens to patients labouring under nervous irritation with chronic debility, from too much professional officiousness. Now and then we meet with cases, in which our choicest stimulants and tonics, however combined and administered, serve but to increase the inquietude and exhaust the strength of our patients. At the same time, there will be great apprehension of danger and perpetual calls upon us, for new and more energetic prescriptions. Under such circumstances, it is the duty of the physician to resist all importunity, and withhold all active medicines. This determination will excite new alarm; but in most cases it will be transient, and within a few days, the condition of the patient will, in all respects, be sensibly improved. During such a period, he should continue his visits, and avail himself of all the aid which can be drawn from moral influences. There is a time to withhold medicines, as well as a time to give them.

